# Epistasis and the sensitivity of phenotypic screens for beta thalassaemia

**DOI:** 10.1186/1475-2875-13-S1-O13

**Published:** 2014-09-22

**Authors:** Bridget Penman, Sunetra Gupta, David Weatherall

**Affiliations:** 1 University of Oxford Zoology Department, Oxford, UK; 2 Weatherall Institute of Molecular Medicine, Oxford, UK

## 

Severe forms of alpha and beta thalassaemia have been estimated to affect approximately 68000 births annually. Individuals who carry thalassaemic genes are protected against death from malaria infection; the global distribution of thalassaemic genes thus matches the historical distribution of malaria.

Screening programmes are a vital tool to counter the thalassaemias by:

(i) identifying individual carriers and allowing them to make informed reproductive choices, and (ii) generating population level gene-frequency estimates, to help ensure the optimal allocation of public health resources. For both of these functions it is vital that the screen performed is suitably sensitive.

One popular first-stage screening option for beta thalassaemia in low-income countries is the One Tube Osmotic Fragility Test (OTOFT). Here we introduce a population genetic framework within which to quantify the likely sensitivity and specificity of the OTOFT in different epidemiological contexts. We demonstrate that the co-occurrence of alpha thalassaemia, and other malaria related erythrocyte poly-morphisms such as Southeast Asian Ovalocytosis and glucose-6-phosphate dehydrogenase deficiency, could reduce the sensitivity of OTOFTs for beta thalassaemia to below 70%. Our results highlight a potential hazard of the widespread application of OTOFTs and emphasize the fact that the public health impact of any single genetic adaptation to malaria cannot be considered in isolation.

